# Supplementary material for: Preclinical and clinical obesity: prevalence, associations to cardiometabolic risk and response to lifestyle intervention in NHANES and the EPIC-Potsdam and TULIP studies
Source: Nat Commun. 2026 Feb 19;17:1935. doi: 10.1038/s41467-026-69738-w (PMC12923867; doi:10.1038/s41467-026-69738-w)
Supplement: Supplementary file 1 — Supplementary Tables [file 41467_2026_69738_MOESM1_ESM.docx]

**Preclinical and clinical obesity: prevalence, associations to cardiometabolic risk and response to lifestyle intervention in NHANES and the EPIC-Potsdam and TULIP studies**

Catarina Schiborn, PhD^1,2^, Frank B. Hu, MD^3,4,5^ Norbert Stefan, MD^2,6,7 §^, Matthias B. Schulze, DrPH^1,2,8 §^*

^§^ equal contribution

* corresponding author

1 Department of Molecular Epidemiology, German Institute of Human Nutrition Potsdam-Rehbruecke, Nuthetal, Germany

2 German Center for Diabetes Research (DZD), München-Neuherberg, Germany

3 Department of Nutrition, Harvard T.H. Chan School of Public Health, Boston, MA, USA

4 Department of Epidemiology, Harvard T.H. Chan School of Public Health, Boston, MA, USA

5 Channing Division of Network Medicine, Department of Medicine, Brigham and Women's Hospital and Harvard Medical School, Boston, MA, USA

6 Department of Internal Medicine IV, University Hospital Tübingen, Tübingen, Germany

7 Institute of Diabetes Research and Metabolic Diseases (IDM) of the Helmholtz Centre Munich, Tübingen, Germany

8 Institute of Nutritional Science, University of Potsdam, Nuthetal, Germany

**Corresponding Author:**

Matthias B. Schulze, DrPH,

Department of Molecular Epidemiology,

German Institute of Human Nutrition Potsdam-Rehbruecke,

Arthur-Scheunert-Allee 114-116,

14558 Nuthetal, Germany

([mschulze@dife.de](mailto:mschulze@dife.de))

**Supplementary Table 1: Characteristics of participants, NHANES 2017-2018 and EPIC-Potsdam**

|  | **NHANES 2017-2018 (n = 5,265)** ^a^ |  | **EPIC-Potsdam (subcohort n = 2,500)** |
| --- | --- | --- | --- |
|  | **Weighted mean or % (95% CI)** |  | **Mean (Std) or % (n)** |
| Age, years | 48.3 (47.2-49.4) |  | 49.8 (9.0) |
| Sex, % |  |  |  |
| Women | 51.9 (50.1-53.7) |  | 61.0 (1,526) |
| Men | 48.1 (46.3-49.9) |  | 39.0 (974) |
| Race and ethnicity, % |  |  | NA |
| Mexican American | 8.8 (5.3-12.3) |  |  |
| Other Hispanic | 7.0 (5.3-8.6) |  |  |
| Non-Hispanic White | 62.2 (56.8-67.7) |  |  |
| Non-Hispanic Black | 11.5 (8.0-14.9) |  |  |
| Non-Hispanic Asian | 5.9 (3.8-7.8) |  |  |
| Other Race - Including Multi-Racial | 4.6 (3.4-5.9) |  |  |
| BMI, kg/m^2^ | 29.8 (29.3-30.3) |  | 26.1 (4.3) |
| Height, cm | 168 (167-168) |  | 168 (9) |
| Waist circumference, cm | 101 (100-102) |  | 86 (13) |
| Waist-to-hip ratio, unitless | 0.93 (0.93-0.94) |  | 0.85 (0.10) |
| Waist -to-height ratio, unitless | 0.60 (0.59-0.61) |  | 0.51 (0.07) |
| DXA total body fat, % | 33.6 (33.2-34.1) |  | NA |

Abbreviations: BMI, body mass index; DXA, dual-energy X-ray absorptiometry; EPIC, European Prospective Investigation into Cancer and Nutrition; NHANES, National Health and Nutrition Examination Survey

^a^ Unweighted number of participants; decimals resulting from multiple imputations were rounded to the nearest integer

**Supplementary Table 2. Fulfillment of anthropometric criteria for definition of clinical obesity in participants with BMI-defined obesity**

|  |  | **DXA measured total body fat >35% in women / >25% in men** | **Elevated waist circumference ^c^** | **Waist-to-hip ratio >0.85 in women / >0.90 in men** | **Waist-to-height ratio >0.5** | **Fulfilling any anthropometric criterion** |  |  |  |  |
| --- | --- | --- | --- | --- | --- | --- | --- | --- | --- | --- |
|  | **No. of participants** ^a^ | **Percent (95% CI or n) ^b^** | | | | |  |  |  |  |
| **NHANES 2017-2028** |  |  |  |  |  |  |  |  |  |  |
| Overall | 2,206 | 91.6 (88.2-95.0) | 96.8 (95.6-98.0) | 92.3 (90.4-94.2) | 99.9 (99.7-100) | 100 (99.7-100) |  |  |  |  |
| BMI categories ^d^ |  |  |  |  |  |  |  |  |  |  |
| Obesity class I | 1,237 | 89.3 (85.1-93.4) | 94.3 (92.4-96.3) | 92.1 (90.1-94.2) | 99.9 (99.5-100) | 99.9 (99.6-100) |  |  |  |  |
| Obesity class II | 614 | 93.2 (88.8-97.6) | 99.3 (97.5-100) | 92.2 (88.8-95.7) | 100 (99.8-100) | 100 (100-100) |  |  |  |  |
| Obesity class III | 483 | 94.9 (88.9-100) | 99.9 (99.6-100) | 92.4 (89.1-95.7) | 100 (99.7-100) | 100 (99.7-100) |  |  |  |  |
| Age groups |  |  |  |  |  |  |  |  |  |  |
| 20-44 years | 820 | 95.0 (92.5-97.6) | 95.0 (92.8-97.2) | 89.0 (85.2-92.7) | 99.9 (99.5-100) | 99.9 (99.5-100) |  |  |  |  |
| 45-64 years | 834 | 92.6 (88.4-96.8) | 97.6 (96.0-99.2) | 94.2 (92.3-96.1) | 100 (99.8-100) | 100 (99.8-100) |  |  |  |  |
| ≥65 years | 552 | 82.3 (69.9-94.6) | 99.2 (98.0-100) | 96.0 (93.6-98.3) | 99.8 (99.3-100) | 100 (100-100) |  |  |  |  |
| Sex |  |  |  |  |  |  |  |  |  |  |
| Women | 1,201 | 89.8 (83.2-96.5) | 99.5 (99.0-100) | 88.3 (85.4-91.2) | 99.9 (99.7-100) | 100 (99.9-100) |  |  |  |  |
| Men | 1,005 | 93.5 (90.9-96.0) | 93.9 (91.6-96.3) | 96.6 (94.5-98.7) | 99.9 (99.5-100) | 99.9 (99.6-100) |  |  |  |  |
| Race |  |  |  |  |  |  |  |  |  |  |
| Mexican American | 347 | 94.6 (88.8-100) | 94.1 (91.6-96.6) | 93.9 (91.1-96.8) | 99.9 (99.5-100) | 100 (100-100) |  |  |  |  |
| Other Hispanic | 199 | 90.2 (82.4-98.1) | 86.6 (80.0-93.2) | 89.9 (84.9-94.8) | 100 (100-100) | 100 (100-100) |  |  |  |  |
| Non-Hispanic White | 794 | 90.9 (86.8-95.0) | 97.9 (96.3-99.4) | 93.7 (91.8-95.6) | 99.9 (99.6-100) | 100 (99.6-100) |  |  |  |  |
| Non-Hispanic Black | 614 | 92.1 (89.9-94.2) | 97.3 (96.1-98.5) | 88.4 (84.3-92.5) | 100 (99.8-100) | 100 (100-100) |  |  |  |  |
| Non-Hispanic Asian | 124 | 90.8 (83.7-97.8) | 98.6 (97.1-100) | 89.1 (81.8-96.3) | 99.8 (99.0-100) | 99.6 (98.7-100) |  |  |  |  |
| Other Race - Including Multi-Racial | 129 | 94.5 (89.3-99.7) | 98.7 (97.2-100) | 86.7 (78.9-94.5) | 100 (100-100) | 100 (100-100) |  |  |  |  |
|  |  |  |  |  |  |  |  |  |  |  |
| **EPIC-Potsdam** |  |  | | | | |  |  |  |  |
| Overall | 394 | NA | 88.3 (348) | 73.1 (288) | 100 (394) | 100 (394) |  |  |  |  |
| BMI categories ^d^ |  |  |  |  |  |  |  |  |  |  |
| Obesity class I | 315 | NA | 85.40 (269) | 73.3 (231) | 100 (315) | 100 (315) |  |  |  |  |
| Obesity class II | 58 | NA | 100 (58) | 81.0 (47) | 100 (58) | 100 (58) |  |  |  |  |
| Obesity class III | 21 | NA | 100 (21) | 47.6 (10) | 100 (21) | 100 (21) |  |  |  |  |
| Age groups |  |  |  |  |  |  |  |  |  |  |
| 35-44 years | 90 | NA | 77.8(70) | 56.7 (51) | 100 (90) | 100 (90) |  |  |  |  |
| ≥45 years | 304 | NA | 91.5 (278) | 78.0 (237) | 100 (304) | 100 (304) |  |  |  |  |
| Sex |  |  |  |  |  |  |  |  |  |  |
| Women | 233 | NA | 90.1 (210) | 55.0 (128) | 100 (233) | 100 (233) |  |  |  |  |
| Men | 161 | NA | 85.7 (138) | 99.4 (160) | 100 (161) | 100 (161) |  |  |  |  |

Abbreviations: BMI, body mass index; DXA, dual-energy X-ray absorptiometry; EPIC, European Prospective Investigation into Cancer and Nutrition; NHANES, National Health and Nutrition Examination Survey

^a^ Unweighted number of participants; decimals resulting from multiple imputations were rounded to the nearest integer in NHANES 2017-2018

^b^ Weighted percent in NHANES 2017-2018 being representative of the total civilian noninstitutionalized US population aged ≥20 years

^c^ in non-Hispanic Asian defined as ≥80 cm for women and ≥90 cm for men and in remaining race/ethnicity defined as ≥88 cm for women and ≥102 for men

^d^ in non-Hispanic Asian obesity class I defined as BMI 27.5-<32.5 kg/m^2^, obesity class II as BMI 32.5-<37.5 kg/m^2^, obesity class III as BMI ≥37.5 kg/m^2^ and in remaining race/ethnicity obesity class I defined as BMI 30.0-34.9 kg/m^2^, obesity class II as BMI 35.0-39.9 kg/m^2^, obesity class III as BMI ≥40.0 kg/m^2^

**Supplementary Table 3. Number of fulfilled clinical criteria for definition of clinical obesity in participants with confirmed obesity (unweighted n = 2,333), NHANES 2017-2018**

| **Number of fulfilled clinical criteria** | **Weighted percent (95% CI)** |
| --- | --- |
| 1 | 18.9 (17.2-20.7) |
| 2 | 20.1 (17.1-23.1) |
| 3 | 16.9 (14.4-19.4) |
| 4 | 12.4 (9.58-15.2) |
| 5 | 7.40 (5.55-9.26) |
| ≥6 | 6.90 (5.25-8.55) |

Abbreviations: NHANES, National Health and Nutrition Examination Survey

**Supplementary Table 4. Fulfillment of individual criteria for definition of clinical obesity in participants with confirmed obesity across sociodemographic subgroups, NHANES 2017-2018**

|  |  | **Fulfillment of clinical criteria, weighted % (95% CI)** | | | | | | | | |
| --- | --- | --- | --- | --- | --- | --- | --- | --- | --- | --- |
|  | **No. of participants ^a^** | **Upper airway** | **Respira-tory** | **Cardio-vascular** | **Meta-bolism** | **Renal** | **Urinary** | **Liver** | **Musculo-skeletal** | **ADL** |
| Overall | 2,333 | 13.1 (10.5-15.6) | 36.9 (34.1-39.7) | 71.1 (67.8-74.4) | 29.0 (25.6-32.3) | 19.3 (17.4-21.3) | 18.4 (16.2-20.6) | 26.0 (22.7-29.4) | 16.6 (13.6-19.7) | 10.6 (8.68-12.6) |
| BMI group ^b^ |  |  |  |  |  |  |  |  |  |  |
| Obesity class I | 1,235 | 6.72 (3.64-9.80) | 33.9 (29.5-38.3) | 67.7 (63.1-72.4) | 28.6 (24.2-33.0) | 17.6 (15.0-20.3) | 15.1 (12.4-17.7) | 14.4 (10.7-18.0) | 15.5 (11.2-19.8) | 7.29 (5.63-8.94) |
| Obesity class II | 614 | 17.9 (12.3-23.4) | 37.1 (31.5-42.6) | 74.3 (68.3-80.3) | 32.6 (26.2-38.9) | 23.4 (19.5-27.3) | 18.7 (13.6-23.9) | 27.2 (22.6-31.8) | 17.5 (12.4-22.6) | 14.5 (9.91-19.1) |
| Obesity class III | 483 | 23.0 (16.1-29.9) | 44.4 (38.0-50.8) | 75.6 (68.8-82.4) | 25.4 (20.2-30.6) | 18.5 (12.9-24.1) | 26.5 (21.2-31.7) | 54.0 (48.2-59.8) | 18.5 (11.7-25.3) | 14.1 (9.72-18.6) |
| Age group |  |  |  |  |  |  |  |  |  |  |
| 20-44 years | 869 | 6.41 (3.50-9.31) | 23.8 (19.5-28.2) | 54.8 (49.7-59.8) | 12.5 (9.97-15.1) | 9.07 (7.42-10.7) | 8.76 (6.98-10.6) | 16.3 (12.1-20.4) | 4.07 (2.12-6.01) | 3.43 (1.57-5.30) |
| 45-64 years | 898 | 19.5 (15.1-23.8) | 42.5 (37.9-47.2) | 79.7 (73.6-85.7) | 33.4 (28.0-38.7) | 18.4 (13.2-23.5) | 19.2 (14.8-23.6) | 28.0 (23.0-33.1) | 21.5 (16.0-27.0) | 10.8 (7.62-14.0) |
| ≥65 years | 566 | 15.1 (10.1-20.1) | 54.6 (49.1-60.1) | 90.2 (84.7-95.8) | 56.3 (49.6-62.9) | 43.4 (36.7-50.2) | 37.9 (30.8-45.0) | 43.5 (36.4-50.6) | 34.8 (29.1-40.4) | 25.9 (18.5-33.3) |
| Sex |  |  |  |  |  |  |  |  |  |  |
| Women | 1,260 | 8.46 (5.47-11.5) | 44.0 (38.7-49.3) | 70.8 (65.2-76.3) | 28.2 (24.2-32.1) | 20.6 (17.3-23.9) | 28.0 (24.7-31.4) | 25.7 (22.2-29.2) | 20.9 (16.8-25.0) | 12.9 (10.5-15.3) |
| Men | 1,073 | 17.9 (13.9-22.0) | 29.4 (25.1-33.6) | 71.5 (67.2-75.8) | 29.8 (25.4-34.3) | 17.9 (14.7-21.1) | 8.17 (5.88-10.5) | 26.4 (21.2-31.6) | 12.2 (9.02-15.3) | 8.22 (5.67-10.8) |
| Race and ethnicity |  |  |  |  |  |  |  |  |  |  |
| Mexican American | 347 | 9.31 (5.42-13.2) | 25.6 (20.7-30.6) | 56.0 (49.8-62.1) | 24.3 (19.4-29.2) | 15.2 (13.0-17.3) | 11.3 (7.92-14.6) | 20.9 (14.7-27.1) | 4.84 (2.40-7.29) | 6.31 (3.42-9.20) |
| Other Hispanic | 199 | 10.7 (4.89-16.6) | 34.9 (27.8-42.0) | 67.1 (58.1-76.1) | 29.7 (21.8-37.6) | 18.0 (11.4-24.5) | 9.68 (5.96-13.4) | 25.8 (18.7-32.9) | 8.42 (3.94-12.9) | 11.6 (5.10-18.0) |
| Non-Hispanic White | 793 | 14.9 (11.7-18.2) | 40.7 (36.6-44.8) | 74.5 (70.0-79.0) | 31.2 (26.9-35.5) | 20.3 (17.3-23.3) | 21.4 (17.8-25.0) | 27.9 (23.0-32.9) | 20.7 (16.4-24.9) | 11.5 (8.70-14.3) |
| Non-Hispanic Black | 614 | 12.3 (9.06-15.4) | 34.4 (29.8-38.9) | 74.4 (68.8-80.0) | 21.5 (17.7-25.4) | 19.1 (15.7-22.4) | 18.1 (15.2-20.9) | 26.6 (23.2-30.1) | 11.7 (9.57-13.8) | 11.3 (7.57-15.0) |
| Non-Hispanic Asian | 250 | 5.11 (1.59-8.63) | 21.4 (15.0-27.8) | 59.3 (51.9-66.7) | 31.0 (23.4-38.7) | 19.0 (14.8-23.2) | 7.58 (5.53-9.62) | 15.4 (12.5-18.2) | 7.12 (3.85-10.4) | 4.69 (2.58-6.81) |
| Other Race - Including Multi-Racial | 129 | 10.1 (1.96-18.2) | 37.4 (26.3-48.4) | 68.2 (54.1-82.4) | 27.7 (13.3-42.2) | 18.5 (10.6-26.4) | 17.7 (9.90-25.6) | 22.1 (13.1-31.0) | 23.4 (9.95-36.8) | 11.3 (4.95-17.6) |

Abbreviations: ADL, activities of daily living; BMI, body mass index; NHANES, National Health and Nutrition Examination Survey

^a^ Unweighted number of participants; Decimals resulting from multiple imputations were rounded to the nearest integer

^b^ in non-Hispanic Asian obesity class I defined as BMI 27.5-32.4 kg/m^2^, obesity class II as BMI 32.5-37.5 kg/m^2^, obesity class III as BMI ≥37.5 kg/m^2^ and in remaining race/ethnicity obesity class I defined as BMI 30.0-34.9 kg/m^2^, obesity class II as BMI 35.0-39.9 kg/m^2^, obesity class III as BMI ≥40.0 kg/m^2^

**Supplementary Table 5. Fulfillment of criteria for definition of clinical obesity in participants without confirmed obesity, NHANES 2017-2018 and EPIC-Potsdam**

|  | **No. of participants ^a^** | **Proportion fulfilling any clinical criterion, % (95% CI or n) ^b^** |
| --- | --- | --- |
| **NHANES** |  |  |
| Overall | 2,932 | 63.3 (60.2-66.4) |
| BMI categories ^c^ |  |  |
| Normal weight | 1,211 | 54.6 (50.2-59.0) |
| Overweight | 1,720 | 70.3 (66.8-73.8) |
| Obesity class I | 1 | 100 (100-100) |
|  |  |  |
| **EPIC Potsdam** |  |  |
| Overall | 1,997 | 72.6 (1450) |
| BMI categories |  |  |
| Normal weight (<25.0 kg/m^2^) | 1,050 | 63.1 (663) |
| Overweight (25.0-29.9 kg/m^2^) | 947 | 83.1 (787) |

Abbreviations: BMI, body mass index; EPIC, European Prospective Investigation into Cancer and Nutrition; NHANES, National Health and Nutrition Examination Survey

^a^ for NHANES 2017-2018: Unweighted number of participants; Decimals resulting from multiple imputations were rounded to the nearest integer

^b^ weighted percent in NHANES 2017-2018 being representative of the total civilian noninstitutionalized US population aged >20 years

^c^ in non-Hispanic Asian normal weight defined as BMI <23 kg/m^2^, overweight as BMI 23.0-27.4 kg/m^2^, obesity class I as BMI 27.5-32.9 kg/m^2^ and in remaining race/ethnicity normal weight defined as BMI <25.0 kg/m^2^, overweight as BMI 25.0-29.9 kg/m^2^, and obesity class I as BMI 30.0-34.9 kg/m^2^

**Supplementary Table 6. Fulfillment of criteria for definition of clinical obesity in participants with confirmed obesity using alternative definitions of the metabolism diagnostic component, NHANES 2017-2018 and EPIC-Potsdam**

|  | **NHANES 2017-2018** | | | **EPIC Potsdam** | | |
| --- | --- | --- | --- | --- | --- | --- |
|  | **No. of participants ^a^** | **Fulfillment of metabolism criterion, weighted % (95% CI)** | **Fulfillment of any criterion, weighted % (95% CI)** | **No. of participants** | **Fulfillment of metabolism criterion, % (n)** | **Fulfillment of any criterion, % (n)** |
| **Metabolism criterion defined as either hyperglycemia (diagnosed T2D, IFG or elevated HbA1c) or dyslipidemia (low HDL-cholesterol and high triglycerides or lipid-lowering medication)*** | | | | | | |
| Overall | 2,333 | 79.7 (77.2-82.2) | 93.5 (91.7-95.3) | 394 | 83.7 (328) ^b^ | 97.0 (382) |
| BMI group ^c^ |  |  |  |  |  |  |
| Obesity I | 1,235 | 77.8 (73.5-82.1) | 92.1 (89.2-95.0) | 315 | 82.4 (258) ^b^ | 96.8 (305) |
| Obesity II | 614 | 80.7 (76.1-85.3) | 94.2 (90.5-98.0) | 58 | 89.7 (52) | 96.6 (56) |
| Obesity III | 483 | 83.4 (73.5-93.3) | 96.0 (93.1-99.0) | 21 | 85.7 (18) | 100 (21) |
|  |  |  |  |  |  |  |
| **Metabolism criterion defined as either diagnosed T2D or hyperglycemia (IFG or elevated HbA1c) with dyslipidemia (low HDL-cholesterol and high triglycerides or lipid-lowering medication)** | | | | | | |
| Overall | 2,333 | 35.3 (32.1-38.5) | 82.8 (80.0-85.6) | 394 | 29.4 (115) ^c^ | 92.9 (366) |
| BMI group ^c^ |  |  |  |  |  |  |
| Obesity I | 1,235 | 33.4 (29.2-37.6) | 79.0 (75.3-82.6) | 315 | 27.2 (85) ^c^ | 92.4 (291) |
| Obesity II | 614 | 39.9 (33.7-46.1) | 84.4 (79.9-88.9) | 58 | 37.9 (22) | 93.1 (54) |
| Obesity III | 483 | 34.5 (27.0-42.0) | 90.6 (86.6-94.6) | 21 | 38.1 (8) | 100 (21) |
|  |  |  |  |  |  |  |
| **Metabolism criterion defined as either diagnosed T2D or hyperglycemia (IFG or elevated HbA1c) with either low HDL-cholesterol or high triglycerides or lipid-lowering medication** | | | | | | |
| Overall | 2,333 | 48.5 (45.5-51.5) | 87.0 (84.5-89.5) | 394 | 55.0 (215) ^c^ | 95.2 (375) |
| BMI group ^c^ |  |  |  |  |  |  |
| Obesity I | 1,235 | 42.6 (38.2-47.0) | 83.0 (79.5-86.5) | 315 | 54.5 (170) ^c^ | 94.6 (298) |
| Obesity II | 614 | 52.9 (48.4-57.3) | 89.3 (84.5-94.2) | 58 | 58.6 (34) | 96.6 (56) |
| Obesity III | 483 | 57.9 (49.3-66.5) | 94.3 (90.7-97.9) | 21 | 52.4 (11) | 100 (21) |
|  |  |  |  |  |  |  |
| **Metabolism criterion defined as either diagnosed T2D or hyperglycemia (IFG or elevated HbA1c) or low HDL-cholesterol or high triglycerides or lipid-lowering medication** | | | | | | |
| Overall | 2,333 | 88.1 (85.5-90.7) | 96.3 (94.8-97.8) | 394 | 91.6 (359) ^b^ | 98.5 (388) |
| BMI group ^c^ |  |  |  |  |  |  |
| Obesity I | 1,235 | 85.3 (81.0-89.6) | 95.0 (92.3-97.7) | 315 | 90.7 (284) ^b^ | 98.4 (310) |
| Obesity II | 614 | 89.9 (85.2-94.7) | 97.4 (95.1-99.6) | 58 | 93.1 (54) | 98.3 (57) |
| Obesity III | 483 | 92.7 (86.5-99.0) | 98.2 (97.0-99.4) | 21 | 100 (21) | 100 (21) |
|  |  |  |  |  |  |  |
| **Metabolism criterion defined as hyperglycemia (self-reported diabetes, impaired fasting glucose or high HbA1c), low HDL-cholesterol levels, and high triglyceride levels** | | | | | | |
| Overall | 2,333 | 12.4 (9.77-15.0) | 82.0 (79.1-84.9) | 394 | 15.4 (60) ^c^ | 92.1 (363) |
| BMI group ^c^ |  |  |  |  |  |  |
| Obesity I | 1,235 | 11.0 (6.77-15.2) | 77.9 (74.0-81.9) | 315 | 12.8 (40) ^c^ | 91.4 (288) |
| Obesity II | 614 | 13.7 (9.61-17.8) | 83.5 (78.7-88.3) | 58 | 25.9 (15) | 93.1 (54) |
| Obesity III | 483 | 14.2 (8.54-19.8) | 90.5 (86.4-94.6) | 21 | 23.8 (5) | 100 (21) |

Abbreviations: BMI, body mass index; EPIC, European Prospective Investigation into Cancer and Nutrition; NHANES, National Health and Nutrition Examination Survey

^a^ Unweighted number of participants; Decimals resulting from multiple imputations were rounded to the nearest integer

^b^ n=2 participants with missing values,

^c^ n=3 participants with missing values,

^d^ in non-Hispanic Asian obesity class I defined as BMI 27.5-32.4 kg/m^2^, obesity class II as BMI 32.5-37.5 kg/m^2^, obesity class III as BMI ≥37.5 kg/m^2^ and in remaining race/ethnicity obesity class I defined as BMI 30.0-34.9 kg/m^2^, obesity class II as BMI 35.0-39.9 kg/m^2^, obesity class III as BMI ≥40.0 kg/m^2^

**Supplementary Table 7. Association of clinical obesity with CVD with adapted reference group, EPIC-Potsdam**

| **Category** | **No of participants /cases** | **HR (95%CI)^a^** |
| --- | --- | --- |
| Non-obese low risk profile ^b^ | 172/6 | Ref |
| Non-obese moderate risk profile ^c^ | 1992/307 | 3.09 (1.22-7.83) |
| Pre-clinical obese | 29/1 | 0.69 (0.07-7.15) |
| Clinical obese | 413/92 | 4.22 (1.63-10.9) |

Abbreviations: EPIC, European Prospective Investigation into Cancer and Nutrition; HR, hazard ratio; CI, confidence interval

^a^ Fully adjusted for age (as underlying time scale) sex, sport and cycling (hours/week), smoking status (never, former, current light <20 units/day, current high >= units/day), educational status (current in training/no certificate/part, skilled worker, professional school, college or higher education/university), alcohol consumption (never, former, current ≤12g (women)/≤24g (men), current >12g (women)/>24g (men)), consumption of whole grain, red meat, and coffee (all in g/day)

^b^ includes non-obese individuals not meeting criteria of any compound of metabolism criterion or any clinical criteria

^c^ includes non-obese individuals meeting any metabolic or clinical criteria

**Supplementary Table 8.** **Anthropometric and metabolic characteristics and fulfillment of criteria of clinical obesity before and after a 9-month lifestyle intervention in adults with BMI ≥30 kg/m^2^ and ≤3% decrease in body weight, Tübingen Lifestyle Intervention Programme**

| **Parameter** | **Baseline** | **Follow-up** | **p-value** |
| --- | --- | --- | --- |
| Gender (males/females) | 29/46 | 29/46 | NA |
| Age (years) | 45 (23-68) | 46 (24-68) | <0.0001 |
| Body weight (kg) | 103.7 (76.8-189.8) | 104.1 (76.7-196.0) | 0.90 |
| BMI (kg/m2) | 35.6 (30.1-51.0) | 35.7 (29.8-53.5) | 0.90 |
| Waist circumference (cm) | 108.2 (88-150) | 107.2 (89-142) | 0.23 |
| Systolic blood pressure (mmHg) | 130.0 (102-168) | 126.5 (90-165) | 0.05 |
| Diastolic blood pressure (mmHg) | 80.7 (54-111) | 79.9 (60-110) | 0.27 |
| eGFR (ml/min/1.73 m^2^) | 87.1 (57-134) | 89.1 (58-144) | 0.13 |
| Triglycerides (mg/dl) | 138.0 (55-871) | 143.2 (39-966) | 0.75 |
| HDL-cholesterol (mg/dl) | 49.1 (27-94) | 46.9 (28-85) | 0.97 |
| Visceral fat _1H-MRT_ (kg) ^a^ | 4.0 (1.1-9.7) | 3.9 (1.0-10.7) | 0.002 |
| Liver fat _MRS_ (%) ^b^ | 8.4 (0.9-30.9) | 6.9 (0.7-23.2) | 0.17 |
| Prediabetes, n (%) | 35 (47) | 37 (49) | <0.0001 |
| Blood pressure-lowering medication, n (%) | 22 (29) | 23 (31) | <0.0001 |
| Lipid-lowering medication (%) | 6 (8) | 5 (7) | <0.0001 |
| Criteria of clinical obesity fulfilled |  |  |  |
| Cardiovascular, n (%) | 46 (61) | 40 (53) | <0.0001 |
| Metabolic, n (%) | 10 (13) | 13 (17) | 0.0008 |
| Renal, n (%) | 4 (5) | 3 (4) | 0.56 |
| Clinical obesity, n (%) | 49 (65) | 46 (61) | <0.0001 |

Data represent unadjusted means (range). Differences between baseline and follow-up were tested using the matched pairs t-test for continuous data and the Fisher's exact test for categorical data. One-sided tests were used for pairwise comparisons of continuous parameters (for increase in age, HDL-C and eGFR and for decrease of all other continuous parameters) and two-sided tests were used for categorical parameters.

MRT: magnetic resonance tomography; MRS: magnetic resonance spectroscopy; eGFR: estimated glomerular filtration rate;

^a^ Available in 62 subjects at baseline and 48 at follow-up.

^b^ At baseline available in 61 and at follow-up in 48 subjects

**Supplementary Table 9. Determinants of remission of clinical obesity during lifestyle intervention** **in participants with ≤3% decrease in body weight, Tübingen Lifestyle Intervention Programme**

| **Covariates** | **Odds ratio** | **95% CI** | **p-value** |
| --- | --- | --- | --- |
| **Univariate logistic regression models** | | | |
| Female sex | 0.43 | 0.09-1.88 | 0.26 |
| Age at baseline (years) | 0.93 | 0.86-1.00 | 0.05 |
| BMI at baseline (kg/m^2^) | 0.88 | 0.71-1.03 | 0.11 |
| Change in weight (kg) | 1.02 | 0.79-1.30 | 0.85 |
| Liver fat at baseline* (%) | 0.96 | 0.84-1.07 | 0.49 |
| Visceral fat at baseline^$^ (kg) | 0.88 | 0.54-1.34 | 0.57 |

OR per increase in one unit of measurement for remission of clinical obesity (n=49) in logistic regression models (Model 1-6). P-values are two-sided based on Likelihood Ratio Chi-Square Test. *Available in 37 subjects. ^$^Available in 40 subjects.

**Supplementary Table 10. Determinants of remission of clinical obesity during lifestyle intervention in participants with >3% decrease in body weight, Tübingen Lifestyle Intervention Programme**

| **Covariates** | **Odds ratio** | **95% CI** | **p-value** |  |
| --- | --- | --- | --- | --- |
| **Univariate logistic regression models** | | | |  |
| Female sex | 1.56 | 0.43-6.51 | 0.50 |  |
| Age (years) | 0.90 | 0.83-0.96 | 0.001 |  |
| BMI at baseline (kg/m^2^) | 1.01 | 0.86-1.20 | 0.83 |  |
| Change in weight (kg) | 1.01 | 0.90-1.16 | 0.87 |  |
| Liver fat at baseline* (%) | 0.77 | 0.60-0.91 | 0.0004 |  |
| Visceral fat at baseline^$^ (kg) | 0.52 | 0.28-0.87 | 0.02 |  |
| **Multivariable logistic regression model^#^** | | | |  |
| Age (years) | 0.91 | 0.82-0.99 | 0.03 |  |
| Liver fat at baseline (%) | 0.80 | 0.61-0.96 | 0.01 |  |
| Visceral fat at baseline (kg) | 0.70 | 0.32-1.35 | 0.30 |  |
|  | | | |  |
| **Forward stepwise regression model** | | | |  |
| **Covariates** | **L-R Chi-square** | **p-value** | **r^2^** |  |
| Age (years) | 12.5 | 0.0004 | 0.26 |  |
| Liver fat at baseline (%) | 5.9 | 0.02 | 0.38 |  |
| Visceral fat at baseline (kg) | 1.08 | 0.30 | 0.41 |  |

OR per increase in one unit of measurement for remission of clinical obesity (n=53) in logistic regression models (Model 1-7) and percentage of remission explained by univariable statistically significant parameters determining remission of clinical obesity, in a forward stepwise regression model.

*Available in 33 subjects. ^#^Available in 33 subjects. r^2^, percentage of remission explained by the forward stepwise regression model.

**Supplementary Table 11.** **Anthropometric and metabolic characteristics and fulfillment of criteria of clinical obesity before and after a 9 month lifestyle intervention in adults with BMI ≥30 kg/m^2^ and ≤5% decrease in body weight, Tübingen Lifestyle Intervention Programme**

| **Parameter** | **Baseline** | **Follow-up** | **p-value** |
| --- | --- | --- | --- |
| Gender (males/females) | 39/65 | 39/65 | NA |
| Age (years) | 46 (23-69) | 47 (24-69) | <0.0001 |
| Body weight (kg) | 102.3 (76.8-189.8) | 101.5 (75.8-196.0) | 0.005 |
| BMI (kg/m^2^) | 35.2 (30.1-51.0) | 35.0 (29.6-53.5) | 0.005 |
| Waist circumference (cm) | 107.7 (85-150) | 105.8 (83-142) | 0.002 |
| Systolic blood pressure (mmHg) | 129.0 (96-168) | 125.3 (90-165) | 0.01 |
| Diastolic blood pressure (mmHg) | 80.0 (54-111) | 78.7 (60-110) | 0.10 |
| eGFR (ml/min/1.73 m^2^) | 87.9 (51-160) | 90.5 (51-194) | 0.05 |
| Triglycerides (mg/dl) | 137.3 (55-871) | 136.6 (39-966) | 0.22 |
| HDL-cholesterol (mg/dl) | 50.2 (27-94) | 48.5 (31-85) | 0.96 |
| Visceral fat _1H-MRT_ (kg) ^a^ | 4.3 (1.2-10.6) | 4.0 (1.1-11.7) | <0.0001 |
| Liver fat _MRS_ (%) ^b^ | 8.1 (0.9-30.9) | 6.6 (0.1-23.2) | 0.09 |
| Prediabetes, n (%) | 46 (44) | 46 (44) | <0.0001 |
| Blood pressure-lowering medication, n (%) | 32 (31) | 31 (30) | <0.0001 |
| Lipid-lowering medication (%) | 7 (7) | 7 (7) | <0.0001 |
| Criteria of clinical obesity fulfilled |  |  |  |
| Cardiovascular, n (%) | 64 (62) | 53 (51) | <0.0001 |
| Metabolic, n (%) | 12 (12) | 15 (14) | 0.0002 |
| Renal, n (%) | 6 (6) | 4 (4) | 0.21 |
| Clinical obesity, n (%) | 68 (65) | 60 (58) | <0.0001 |

Data represent unadjusted means (range). Differences between baseline and follow-up were tested using the matched pairs *t* test for continuous data and the Fisher's exact test for categorical data. One-sided tests were used for pairwise comparisons of continuous parameters (for increase in age, HDL-C and eGFR and for decrease of all other continuous parameters) and two-sided tests were used for categorical parameters.

MRT: magnetic resonance tomography; MRS: magnetic resonance spectroscopy; eGFR: estimated glomerular filtration rate;

^a^ Available in 84 subjects at baseline and 67 at follow-up. ^b^ At baseline available in 82 and at follow-up in 65 subjects

**Supplementary Table 12 Anthropometric and metabolic characteristics and fulfillment of criteria of clinical obesity before and after a 9-month lifestyle intervention in adults with BMI ≥30 kg/m^2^, and >5% decrease in body weight, Tübingen Lifestyle Intervention Programme**

| **Parameter** | **Baseline** | **Follow-up** | **p-value** |
| --- | --- | --- | --- |
| Gender (males/females) | 20/26 | 20/26 | NA |
| Age (years) | 49 (25-69) | 49 (25-70) | <0.0001 |
| Body weight (kg) | 102.3 (66.5-142.7) | 92.5 (62.7-133.5) | <0.0001 |
| BMI (kg/m^2^) | 35.2 (30.1-45.7) | 31.8 (26.1-40.2) | <0.0001 |
| Waist circumference (cm) | 111.0 (93-135) | 102.8 (85-134) | <0.0001 |
| Systolic blood pressure (mmHg) | 133.3 (100-181) | 126.6 (95-153) | 0.002 |
| Diastolic blood pressure (mmHg) | 79.9 (49-108) | 75.4 (52-99) | 0.008 |
| eGFR (ml/min/1.73 m^2^) | 85.2 (58-128) | 86.3 (59-128) | 0.32 |
| Triglycerides (mg/dl) | 156.5 (41-857) | 116.8 (49-383) | 0.0007 |
| HDL-cholesterol (mg/dl) | 49.1 (33-81) | 48.0 (33-67) | 0.84 |
| Visceral fat _1H-MRT_ (kg) ^a^ | 4.5 (1.1-10.1) | 3.3 (0.8-7.7) | <0.0001 |
| Liver fat _MRS_ (%) ^b^ | 10.3 (0.8-30.1) | 4.0 (0.1-13.4) | 0.0002 |
| Prediabetes, n (%) | 28 (61) | 13 (28) | 0.05 |
| Blood pressure-lowering medication, n (%) | 20 (43) | 19 (41) | <0.0001 |
| Lipid-lowering medication (%) | 4 (9) | 6 (13) | 0.005 |
| Criteria of clinical obesity fulfilled |  |  |  |
| Cardiovascular, n (%) | 33 (72) | 29 (37) | <0.0001 |
| Metabolic, n (%) | 8 (17) | 3 (7) | 0.004 |
| Renal, n (%) | 1 (2) | 1 (2) | 1.00 |
| Clinical obesity, n (%) | 34 (74) | 29 (63) | 0.0002 |

Data represent unadjusted means (range). Differences between baseline and follow-up were tested using the matched pairs *t* test for continuous data and the Fisher's exact test for categorical data. One-sided tests were used for pairwise comparisons of continuous parameters (for increase in age, HDL-C and eGFR and for decrease of all other continuous parameters) and two-sided tests were used for categorical parameters.

MRT: magnetic resonance tomography; MRS: magnetic resonance spectroscopy; eGFR: estimated glomerular filtration rate;

^a^ Available in 37 subjects at baseline and 32 at follow-up. ^b^ At baseline available in 33 and at follow-up in 28 subjects

**Supplementary Table 13. Determinants of remission of clinical obesity during lifestyle intervention in participants with ≤5% decrease in body weight, Tübingen Lifestyle Intervention Programme**

| **Covariates** | **Odds ratio** | **95% CI** | **p-value** |
| --- | --- | --- | --- |
| **Univariate logistic regression models** | | | |
| Female sex | 0.96 | 0.31-3.21 | 0.94 |
| Age at baseline (years) | 0.94 | 0.88-0.99 | 0.02 |
| BMI at baseline (kg/m^2^) | 0.91 | 0.78-1.03 | 0.13 |
| Change in weight (kg) | 0.91 | 0.74-1.10 | 0.37 |
| Liver fat at baseline* (%) | 0.94 | 0.84-1.03 | 0.19 |
| Visceral fat at baseline^$^ (kg) | 0.67 | 0.42-0.98 | 0.04 |
| **Multivariable logistic regression model^#^** | | | |
| Age (years) | 0.96 | 0.90-1.02 | 0.22 |
| Visceral fat at baseline (kg) | 0.72 | 0.45-1.07 | 0.11 |
|  |  |  |  |
| **Forward stepwise regression model** | | | |
| **Covariates** | **L-R Chi-square** | **p-value** | **r^2^** |
| Age (years) | 4.3 | 0.04 | 0.07 |
| Visceral fat at baseline (kg) | 1.5 | 0.22 | 0.09 |

OR per increase in one unit of measurement for remission of clinical obesity (n=34) in logistic regression models (Model 1-6) and percentage of remission explained by univariable statistically significant parameters determining remission of clinical obesity, in a forward stepwise regression model *Available in 54 subjects. ^$^Available in 56 subjects. ^#^Available in 56 subjects. r^2^, percentage of remission explained by the forward stepwise regression model.

**Supplementary Table 14. Determinants of remission of clinical obesity during lifestyle intervention in participants with >5% decrease in body weight, Tübingen Lifestyle Intervention Programme**

| **Covariates** | **Odds ratio** | **95% CI** | **p-value** |
| --- | --- | --- | --- |
| **Univariate logistic regression models** | | | |
| Female sex | 0.78 | 0.14-4.66 | 0.78 |
| Age at baseline (years) | 0.89 | 0.80-0.97 | 0.008 |
| BMI at baseline (kg/m^2^) | 1.06 | 0.84-1.33 | 0.61 |
| Change in weight (kg) | 0.94 | 0.80-1.11 | 0.43 |
| Liver fat at baseline* (%) | 0.60 | 0.3-0.85 | 0.0002 |
| Visceral fat at baseline^$^ (kg) | 0.71 | 0.34-1.34 | 0.30 |
| **Multivariable logistic regression model^#^** | | | |
| Age (years) | 0.91 | 0.75-1.02 | 0.11 |
| Liver fat at baseline (%) | 0.58 | 0.27-0.86 | 0.0009 |
|  | | | |
| **Forward stepwise regression model** | | | |
| **Covariates** | **L-R Chi-square** | **p-value** | **r^2^** |
| Liver fat at baseline (%) | 13.5 | 0.0002 | 0.49 |
| Age at baseline (years) | 2.6 | 0.11 | 0.58 |

OR per increase in one unit of measurement for remission of clinical obesity (n=68) in logistic regression models (Model 1-6) and percentage of remission explained by univariable statistically significant parameters determining remission of clinical obesity, in a forward stepwise regression model. *Available in 22 subjects. ^$^Available in 25 subjects. ^#^Available in 22 subjects. r^2^, percentage of remission explained by the forward stepwise regression model.

**Supplementary Table 15. Application of criteria for clinical obesity in NHANES 2017-2018**

| **NHANES measure of organ, tissue, or body function** | **Definition** | **NHANES data items used** |
| --- | --- | --- |
| **Respiratory System** |  |  |
| Breathlessness | yes | [CDQ010 - Shortness of breath on stairs/inclines](https://wwwn.cdc.gov/Nchs/Data/Nhanes/Public/2017/DataFiles/CDQ_J.htm#CDQ010) |
| **Upper Airways** |  |  |
| STOP-Bang questionnaire ^a^ | STOP-Bang sum points >=5 |  |
| Snoring | 3-4 nights a week or more | [SLQ030 - How often do you snore?](https://wwwn.cdc.gov/Nchs/Data/Nhanes/Public/2017/DataFiles/SLQ_J.htm#SLQ030) |
| Snot or stop breathing | 3-4 nights a week or more | [SLQ040 - How often do you snort or stop breathing](https://wwwn.cdc.gov/Nchs/Data/Nhanes/Public/2017/DataFiles/SLQ_J.htm#SLQ040) |
| Trouble Sleeping | yes | [SLQ050 - Ever told doctor had trouble sleeping?](https://wwwn.cdc.gov/Nchs/Data/Nhanes/Public/2017/DataFiles/SLQ_J.htm#SLQ050) |
| Daytime sleepiness | 5-15 times a month or more | [SLQ120 - How often feel overly sleepy during day?](https://wwwn.cdc.gov/Nchs/Data/Nhanes/Public/2017/DataFiles/SLQ_J.htm#SLQ120) |
| Age | >50 years | [RIDAGEYR - Age in years at screening](https://wwwn.cdc.gov/Nchs/Data/Nhanes/Public/2017/DataFiles/DEMO_J.htm#RIDAGEYR) |
| Gender | male | [RIAGENDR - Gender](https://wwwn.cdc.gov/Nchs/Data/Nhanes/Public/2017/DataFiles/DEMO_J.htm#RIAGENDR) |
| BMI (Body Mass Index) | >35 kg/m^2^ | [BMXBMI - Body Mass Index (kg/m**2)](https://wwwn.cdc.gov/Nchs/Data/Nhanes/Public/2017/DataFiles/BMX_J.htm#BMXBMI) |
| **Cardiovascular System ^b^** |  |  |
| Hypertensive medication | yes | [BPQ040A - Taking prescription for hypertension](https://wwwn.cdc.gov/Nchs/Data/Nhanes/Public/2017/DataFiles/BPQ_J.htm#BPQ040A) |
| Blood pressure measurements |  |  |
| systolic | average of 2nd and 3rd reading >130 mmHg | [BPXOSY2 - Systolic - 2nd oscillometric reading](https://wwwn.cdc.gov/Nchs/Data/Nhanes/Public/2017/DataFiles/BPXO_J.htm#BPXOSY2) |
|  |  | [BPXOSY3 - Systolic - 3rd oscillometric reading](https://wwwn.cdc.gov/Nchs/Data/Nhanes/Public/2017/DataFiles/BPXO_J.htm#BPXOSY3) |
| diastolic | average of 2nd and 3rd reading >80 mmHg | [BPXODI2 - Diastolic - 2nd oscillometric reading](https://wwwn.cdc.gov/Nchs/Data/Nhanes/Public/2017/DataFiles/BPXO_J.htm#BPXODI2) |
|  |  | [BPXODI3 - Diastolic - 3rd oscillometric reading](https://wwwn.cdc.gov/Nchs/Data/Nhanes/Public/2017/DataFiles/BPXO_J.htm#BPXODI3) |
| Breathlessness | yes | [CDQ010 - Shortness of breath on stairs/inclines](https://wwwn.cdc.gov/Nchs/Data/Nhanes/Public/2017/DataFiles/CDQ_J.htm#CDQ010) |
| Self-reported prevalent diseases |  |  |
| Heart failure | yes | [MCQ160b - Ever told had congestive heart failure](https://wwwn.cdc.gov/Nchs/Data/Nhanes/Public/2017/DataFiles/MCQ_J.htm#MCQ160b) |
| Coronary heart disease | yes | [MCQ160c - Ever told you had coronary heart disease](https://wwwn.cdc.gov/Nchs/Data/Nhanes/Public/2017/DataFiles/MCQ_J.htm#MCQ160c) |
| Heart attack | yes | [MCQ160e - Ever told you had heart attack](https://wwwn.cdc.gov/Nchs/Data/Nhanes/Public/2017/DataFiles/MCQ_J.htm#MCQ160e) |
| Stroke | yes | [MCQ160f - Ever told you had a stroke](https://wwwn.cdc.gov/Nchs/Data/Nhanes/Public/2017/DataFiles/MCQ_J.htm#MCQ160f) |
| **Metabolism ^c^** |  |  |
| Self-reported diabetes | yes | [DIQ010 - Doctor told you have diabetes](https://wwwn.cdc.gov/Nchs/Data/Nhanes/Public/2017/DataFiles/DIQ_J.htm#DIQ010) |
| Hyperglycemia |  |  |
| HbA1c | ≥5.7 % | [LBXGH - Glycohemoglobin (%)](https://wwwn.cdc.gov/Nchs/Data/Nhanes/Public/2017/DataFiles/GHB_J.htm#LBXGH) |
| Fasting plasma glucose | ≥100 mg/dl | [LBXGLU - Fasting Glucose (mg/dL)](https://wwwn.cdc.gov/Nchs/Data/Nhanes/Public/2017/DataFiles/GLU_J.htm#LBXGLU) |
| Dyslipidemia |  |  |
| Plasma triglycerides | >150 mg/dl | [LBXTR - Triglyceride (mg/dL)](https://wwwn.cdc.gov/Nchs/Data/Nhanes/Public/2017/DataFiles/TRIGLY_J.htm#LBXTR) |
| Plasma HDL-cholesterol | ≤ 40 mg/dl M; <50 mg/dl F | [LBDHDD - Direct HDL-Cholesterol (mg/dL)](https://wwwn.cdc.gov/Nchs/Data/Nhanes/Public/2017/DataFiles/HDL_J.htm#LBDHDD) |
| **Renal ^b^** |  |  |
| Self-reported dialysis | yes | [KIQ025 - Received dialysis in past 12 months?](https://wwwn.cdc.gov/Nchs/Data/Nhanes/Public/2017/DataFiles/KIQ_U_J.htm#KIQ025) |
| Urinary Albumin-Creatinine Ratio (UACR) | ≥30 mg/mmol | [URDACT - Albumin creatinine ratio (mg/g)](https://wwwn.cdc.gov/Nchs/Data/Nhanes/Public/2017/DataFiles/ALB_CR_J.htm#URDACT) |
| Estimated Glomerular Filtration Rate (eGFR, CKD-EPI Equation) | <60 mL/min per 1.73 m^2^ | [LBXSCR - Creatinine, refrigerated serum (mg/dL)](https://wwwn.cdc.gov/Nchs/Data/Nhanes/Public/2017/DataFiles/BIOPRO_J.htm#LBXSCR) |
|  |  | [RIDAGEYR - Age in years at screening](https://wwwn.cdc.gov/Nchs/Data/Nhanes/Public/2017/DataFiles/DEMO_J.htm#RIDAGEYR) |
|  |  | [RIAGENDR - Gender](https://wwwn.cdc.gov/Nchs/Data/Nhanes/Public/2017/DataFiles/DEMO_J.htm#RIAGENDR) |
|  |  | [RIDRETH1 - Race/Hispanic origin](https://wwwn.cdc.gov/Nchs/Data/Nhanes/Public/2017/DataFiles/DEMO_J.htm#RIDRETH1) |
| **Urinary System ^b^** |  |  |
| Urinary leakage | a few times a week or more | [KIQ005 - How often have urinary leakage?](https://wwwn.cdc.gov/Nchs/Data/Nhanes/Public/2017/DataFiles/KIQ_U_J.htm#KIQ005) |
| Urinary leakage during physical activities | a few times a week or more | [KIQ042 - Leak urine during physical activities?](https://wwwn.cdc.gov/Nchs/Data/Nhanes/Public/2017/DataFiles/KIQ_U_J.htm#KIQ042) |
|  |  | [KIQ430 - How frequently does this occur?](https://wwwn.cdc.gov/Nchs/Data/Nhanes/Public/2017/DataFiles/KIQ_U_J.htm#KIQ430) |
| Urinated before reaching the toilet | a few times a week or more | [KIQ044 - Urinated before reaching the toilet?](https://wwwn.cdc.gov/Nchs/Data/Nhanes/Public/2017/DataFiles/KIQ_U_J.htm#KIQ044) |
|  |  | [KIQ450 - How frequently does this occur?](https://wwwn.cdc.gov/Nchs/Data/Nhanes/Public/2017/DataFiles/KIQ_U_J.htm#KIQ450) |
| Leak urine during nonphysical activities | a few times a week or more | [KIQ046 - Leak urine during nonphysical activities](https://wwwn.cdc.gov/Nchs/Data/Nhanes/Public/2017/DataFiles/KIQ_U_J.htm#KIQ046) |
|  |  | [KIQ470 - How frequently does this occur?](https://wwwn.cdc.gov/Nchs/Data/Nhanes/Public/2017/DataFiles/KIQ_U_J.htm#KIQ470) |
| **Liver ^b^** |  |  |
| FIB-4 score | ≥2.67 | [LBXSASSI - Aspartate Aminotransferase (AST) (U/L)](https://wwwn.cdc.gov/Nchs/Data/Nhanes/Public/2017/DataFiles/BIOPRO_J.htm#LBXSASSI) |
|  |  | [LBXSATSI - Alanine Aminotransferase (ALT) (U/L)](https://wwwn.cdc.gov/Nchs/Data/Nhanes/Public/2017/DataFiles/BIOPRO_J.htm#LBXSATSI) |
|  |  | [LBXPLTSI - Platelet count (1000 cells/uL)](https://wwwn.cdc.gov/Nchs/Data/Nhanes/Public/2017/DataFiles/CBC_J.htm#LBXPLTSI) |
| NAFLD (Non-Alcoholic Fatty Liver Disease) Fibrosis Score | ≥0.672 | [LBXSATSI - Alanine Aminotransferase (ALT) (U/L)](https://wwwn.cdc.gov/Nchs/Data/Nhanes/Public/2017/DataFiles/BIOPRO_J.htm#LBXSATSI) |
|  |  | [LBXSASSI - Aspartate Aminotransferase (AST) (U/L)](https://wwwn.cdc.gov/Nchs/Data/Nhanes/Public/2017/DataFiles/BIOPRO_J.htm#LBXSASSI) |
|  |  | [LBXSAL - Albumin, refrigerated serum (g/dL)](https://wwwn.cdc.gov/Nchs/Data/Nhanes/Public/2017/DataFiles/BIOPRO_J.htm#LBXSAL) |
|  |  | [LBXPLTSI - Platelet count (1000 cells/uL)](https://wwwn.cdc.gov/Nchs/Data/Nhanes/Public/2017/DataFiles/CBC_J.htm#LBXPLTSI) |
|  |  | [BMXBMI - Body Mass Index (kg/m**2)](https://wwwn.cdc.gov/Nchs/Data/Nhanes/Public/2017/DataFiles/BMX_J.htm#BMXBMI) |
|  |  | [RIDAGEYR - Age in years at screening](https://wwwn.cdc.gov/Nchs/Data/Nhanes/Public/2017/DataFiles/DEMO_J.htm#RIDAGEYR) |
|  |  | [DIQ010 - Doctor told you have diabetes](https://wwwn.cdc.gov/Nchs/Data/Nhanes/Public/2017/DataFiles/DIQ_J.htm#DIQ010) |
| US/Fibroscan | ≥7.9 kPa | [LUXSMED - Median stiffness (E), kilopascals (kPa)](https://wwwn.cdc.gov/Nchs/Data/Nhanes/Public/2017/DataFiles/LUX_J.htm#LUXSMED) |
| **Musculoskeletal System** |  |  |
| Self-reported prevalent conditions | Osteoarthritis or degenerative arthritis | [MCQ160a - Doctor ever said you had arthritis](https://wwwn.cdc.gov/Nchs/Data/Nhanes/Public/2017/DataFiles/MCQ_J.htm#MCQ160a) |
|  |  | [MCQ195 - Which type of arthritis was it?](https://wwwn.cdc.gov/Nchs/Data/Nhanes/Public/2017/DataFiles/MCQ_J.htm#MCQ195) |
| **Activities of Daily Living (ADL) ^b^** |  |  |
| Limitations of activities | Caused by weight problems | [PFQ049 - Limitations keeping you from working](https://wwwn.cdc.gov/Nchs/Data/Nhanes/Public/2017/DataFiles/PFQ_J.htm#PFQ049) |
|  |  | [PFQ051 - Limited in amount of work you can do](https://wwwn.cdc.gov/Nchs/Data/Nhanes/Public/2017/DataFiles/PFQ_J.htm#PFQ051) |
|  |  | [PFQ054 - Need special equipment to walk](https://wwwn.cdc.gov/Nchs/Data/Nhanes/Public/2017/DataFiles/PFQ_J.htm#PFQ054) |
|  |  | [PFQ061B - Difficulty walking for a quarter mile](https://wwwn.cdc.gov/Nchs/Data/Nhanes/Public/2017/DataFiles/PFQ_J.htm#PFQ061B) |
|  |  | [PFQ061C - Difficulty walking up ten stairs](https://wwwn.cdc.gov/Nchs/Data/Nhanes/Public/2017/DataFiles/PFQ_J.htm#PFQ061C) |
|  |  | [PFQ061D - Difficulty stooping, crouching, kneeling](https://wwwn.cdc.gov/Nchs/Data/Nhanes/Public/2017/DataFiles/PFQ_J.htm#PFQ061D) |
|  |  | [PFQ061E - Difficulty lifting or carrying](https://wwwn.cdc.gov/Nchs/Data/Nhanes/Public/2017/DataFiles/PFQ_J.htm#PFQ061E) |
|  |  | [PFQ061F - Difficulty doing house chores](https://wwwn.cdc.gov/Nchs/Data/Nhanes/Public/2017/DataFiles/PFQ_J.htm#PFQ061F) |
|  |  | [PFQ061G - Difficulty preparing meals](https://wwwn.cdc.gov/Nchs/Data/Nhanes/Public/2017/DataFiles/PFQ_J.htm#PFQ061G) |
|  |  | [PFQ061H - Difficulty walking between rooms](https://wwwn.cdc.gov/Nchs/Data/Nhanes/Public/2017/DataFiles/PFQ_J.htm#PFQ061H) |
|  |  | [PFQ061I - Difficulty standing up from armless chair](https://wwwn.cdc.gov/Nchs/Data/Nhanes/Public/2017/DataFiles/PFQ_J.htm#PFQ061I) |
|  |  | [PFQ061J - Difficulty getting in and out of bed](https://wwwn.cdc.gov/Nchs/Data/Nhanes/Public/2017/DataFiles/PFQ_J.htm#PFQ061J) |
|  |  | [PFQ063A - Health problem causing difficulty](https://wwwn.cdc.gov/Nchs/Data/Nhanes/Public/2017/DataFiles/PFQ_J.htm#PFQ063A) |
|  |  | [PFQ063B - Health problem causing difficulty](https://wwwn.cdc.gov/Nchs/Data/Nhanes/Public/2017/DataFiles/PFQ_J.htm#PFQ063B) |
|  |  | [PFQ063C - Health problem causing difficulty](https://wwwn.cdc.gov/Nchs/Data/Nhanes/Public/2017/DataFiles/PFQ_J.htm#PFQ063C) |
|  |  | [PFQ063D - Health problem causing difficulty](https://wwwn.cdc.gov/Nchs/Data/Nhanes/Public/2017/DataFiles/PFQ_J.htm#PFQ063D) |
|  |  | [PFQ063E - Health problem causing difficulty](https://wwwn.cdc.gov/Nchs/Data/Nhanes/Public/2017/DataFiles/PFQ_J.htm#PFQ063E) |
| **Central Nervous System** |  |  |
| No measures identified | NA | NA |
| **Reproductive System** |  |  |
| No measures identified | NA | NA |
| **Lymphatic System** |  |  |
| No measures identified | NA | NA |

^a^ Original instrument additionally includes neck size as a criterion, which was not assessed in NHANES 2017-2018, and could thus not be included

^b^ Fulfilled if any of the defined criteria is met

^c^ Fulfilled in case of self-reported diabetes or hyperglycemia (increased HbA1c or fasting glucose levels) in combination with increased triglycerides and decreased HDL-levels

**Supplementary Table 16. Absolute and proportional missingness of parameter relevant for analyses or to be used as auxiliary parameters in the imputation selectively shown** **for individuals ≥20 years who attended the medical examination, NHANES 2017-2018**

| **Variable** | **Parameter** | **N Missing** | **Proportional missingness in examination sample, %** |
| --- | --- | --- | --- |
| URXUMS | Albumin, urine (mg/L) | 138 | 1.6 |
| URDACT | Albumin creatinine ratio (mg/g) | 138 | 1.6 |
| LBXSATSI | Alanine Aminotransferase (ALT) (U/L) | 350 | 4.0 |
| LBXSAL | Albumin, refrigerated serum (g/dL) | 348 | 4.0 |
| LBXSASSI | Aspartate Aminotransferase (AST) (U/L) | 363 | 4.2 |
| LBXSBU | Blood Urea Nitrogen (mg/dL) | 351 | 4.0 |
| LBXSCR | Creatinine, refrigerated serum (mg/dL) | 349 | 4.0 |
| LBXSGL | Glucose, refrigerated serum (mg/dL) | 351 | 4.0 |
| LBXSGTSI | Gamma Glutamyl Transferase (GGT) (IU/L) | 350 | 4.0 |
| LBDSCHSI | Cholesterol, refrigerated serum (mmol/L) | 349 | 4.0 |
| LBXSTR | Triglycerides, refrig serum (mg/dL) | 351 | 4.0 |
| LBXSUA | Uric acid (mg/dL) | 351 | 4.0 |
| BMXWT | Weight (kg) | 80 | 0.9 |
| BMXHT | Standing Height (cm) | 80 | 0.9 |
| BMXBMI | Body Mass Index (kg/m**2) | 90 | 1.0 |
| BMXWAIST | Waist Circumference (cm) | 328 | 3.8 |
| BMXHIP | Hip Circumference (cm) | 316 | 3.6 |
| BPQ020 | Ever told you had high blood pressure | 0 | 0.0 |
| BPQ040A | Taking prescription for hypertension | 0 | 0.0 |
| BPQ050A | Now taking prescribed medicine for HBP | 177 | 2.0 |
| BPQ090D | Told to take prescriptn for cholesterol | 1126 | 12.9 |
| BPQ100D | Now taking prescribed medicine | 0 | 0.0 |
| BPXOSY1 | Systolic - 1st oscillometric reading | 692 | 8.0 |
| BPXODI1 | Diastolic - 1st oscillometric reading | 692 | 8.0 |
| BPXOSY2 | Systolic - 2nd oscillometric reading | 702 | 8.1 |
| BPXODI2 | Diastolic - 2nd oscillometric reading | 702 | 8.1 |
| BPXOSY3 | Systolic - 3rd oscillometric reading | 713 | 8.2 |
| BPXODI3 | Diastolic - 3rd oscillometric reading | 713 | 8.2 |
| LBXPLTSI | Platelet count (1000 cells/uL) | 237 | 2.7 |
| CDQ010 | Shortness of breath on stairs/inclines | 1589 | 18.3 |
| RIAGENDR | Gender | 0 | 0.0 |
| RIDAGEYR | Age in years at screening | 0 | 0.0 |
| RIDRETH1 | Race/Hispanic origin | 0 | 0.0 |
| RIDRETH3 | Race/Hispanic origin w/ NH Asian | 0 | 0.0 |
| DMDBORN4 | Country of birth | 0 | 0.0 |
| DMDEDUC2 | Education level - Adults 20+ | 12 | 0.1 |
| DIQ010 | Doctor told you have diabetes | 0 | 0.0 |
| DIQ050 | Taking insulin now | 0 | 0.0 |
| DIQ070 | Take diabetic pills to lower blood sugar | 0 | 0.0 |
| DXDTOPF | Total Percent Fat | 3015 | 34.6 |
| LBXGH | Glycohemoglobin (%) | 247 | 2.8 |
| LBXGLU | Fasting Glucose (mg/dL) | 2830 | 32.5 |
| LBDHDD | Direct HDL-Cholesterol (mg/dL) | 328 | 3.8 |
| KIQ022 | Ever told you had weak/failing kidneys? | 0 | 0.0 |
| KIQ025 | Received dialysis in past 12 months? | 0 | 0.0 |
| KIQ005 | How often have urinary leakage? | 386 | 4.4 |
| KIQ010 | How much urine lose each time? | 386 | 4.4 |
| KIQ042 | Leak urine during physical activities? | 387 | 4.4 |
| KIQ430 | How frequently does this occur? | 389 | 4.5 |
| KIQ044 | Urinated before reaching the toilet? | 388 | 4.5 |
| KIQ450 | How frequently does this occur? | 388 | 4.5 |
| KIQ046 | Leak urine during nonphysical activities | 389 | 4.5 |
| KIQ470 | How frequently does this occur? | 389 | 4.5 |
| KIQ050 | How much did urine leakage bother you? | 390 | 4.5 |
| KIQ052 | How much were daily activities affected? | 390 | 4.5 |
| KIQ480 | How many times urinate in night? | 389 | 4.5 |
| LUXSMED | Median stiffness (E), kilopascals (kPa) | 395 | 4.5 |
| LUXSIQR | Stiffness E interquartile range (IQRe) | 387 | 4.4 |
| LUXCAPM | Median CAP, decibels per meter (dB/m) | 396 | 4.5 |
| LUXCPIQR | CAP interquartile range (IQRc) | 402 | 4.6 |
| MCQ160A | Doctor ever said you had arthritis | 0 | 0.0 |
| MCQ195 | Which type of arthritis was it? | 0 | 0.0 |
| MCQ160B | Ever told had congestive heart failure | 0 | 0.0 |
| MCQ160C | Ever told you had coronary heart disease | 0 | 0.0 |
| MCQ160D | Ever told you had angina/angina pectoris | 0 | 0.0 |
| MCQ160E | Ever told you had heart attack | 0 | 0.0 |
| MCQ160F | Ever told you had a stroke | 0 | 0.0 |
| MCQ160L | Ever told you had any liver condition | 0 | 0.0 |
| MCQ170L | Do you still have a liver condition | 4981 | 57.2 |
| MCQ500 | Ever told you had any liver condition | 5265 | 60.5 |
| PFQ049 | Limitations keeping you from working | 0 | 0.0 |
| PFQ051 | Limited in amount of work you can do | 0 | 0.0 |
| PFQ054 | Need special equipment to walk | 0 | 0.0 |
| PFQ061B | Difficulty walking for a quarter mile | 682 | 7.8 |
| PFQ061C | Difficulty walking up ten stairs | 682 | 7.8 |
| PFQ061D | Difficulty stooping, crouching, kneeling | 0 | 0.0 |
| PFQ061E | Difficulty lifting or carrying | 0 | 0.0 |
| PFQ061F | Difficulty doing house chores | 0 | 0.0 |
| PFQ061G | Difficulty preparing meals | 0 | 0.0 |
| PFQ061H | Difficulty walking between rooms | 0 | 0.0 |
| PFQ061I | Difficulty standingup from armless chair | 0 | 0.0 |
| PFQ061J | Difficulty getting in and out of bed | 0 | 0.0 |
| PFQ063A | Health problem causing difficulty | 5 | 0.1 |
| PFQ063B | Health problem causing difficulty | 482 | 5.5 |
| PFQ063C | Health problem causing difficulty | 802 | 9.2 |
| PFQ063D | Health problem causing difficulty | 1050 | 12.1 |
| PFQ063E | Health problem causing difficulty | 1272 | 14.6 |
| RHQ074 | Tried for a year to become pregnant? | 1137 | 13.1 |
| RHQ076 | Seen a DR b/c unable to become pregnant? | 1137 | 13.1 |
| SLQ030 | How often do you snore? | 0 | 0.0 |
| SLQ040 | How often do you snort or stop breathing | 0 | 0.0 |
| SLQ050 | Ever told doctor had trouble sleeping? | 0 | 0.0 |
| SLQ120 | How often feel overly sleepy during day? | 0 | 0.0 |
| LBXTC | Total Cholesterol (mg/dL) | 328 | 3.8 |
| LBXTR | Triglyceride (mg/dL) | 2872 | 33.0 |
| LBDLDLN | LDL-Cholesterol, NIH equation 2 (mg/dL) | 2879 | 33.1 |

All listed variables were included in the imputation model. Missingness due to previous filter questions is not included. Imputations were carried out in the overall medical examination sample, missingness is selectively shown for individuals ≥20 years who attended the medical examination
